# Real-Time Voltammetric Assay of Lead Ion in Biological Cell Systems

**DOI:** 10.5487/TR.2009.25.4.231

**Published:** 2009-12-30

**Authors:** Suw Young Ly

**Affiliations:** Biosensor Research Institute, Seoul National University of Technology, 172, Gongreung 2-dong Nowon-gu, Seoul, 139-743 Korea

**Keywords:** Lead, Voltammetry, Tissue

## Abstract

Trace lead detection for cyclic voltammetry (CV) and square-wave (SW) stripping voltammetry was performed using mercury immobilized onto a carbon nanotube electrode (HNPE). Using the characteristics of mercury and the catalytic carbon nanotube structure, a modified technique, the 0.45 µg/l detection limit of lead ion was attained. The developed method can be applied to pond water, fish tissue, plant tissue, and *in vivo* direct assay.

## INTRODUCTION

In *in vivo* biological systems, absorbed lead is related to the medicinal risk facts of organic and neuro disease (Vagn *et al.*, 2001). Trace Pb(II) can be absorbed through the skin, contaminated food, cigarettes (Lauralynn *et al.*, 2001) and water. Pb(II) exposure remains significant for many populations worldwide despite the major restrictions on certain uses of lead (e.g., as gasoline additives) (Ellen *et al.*, 2003). Trace detection methods are important in biological and environmental (Marıa *et al.*, 2004) monitoring controls (Joseph *et al.,* 2001). For these reasons, some methods of detecting lead have been developed. These include the spectrophotometric graphite furnace atomic absorption spectrometry (Huge *et al.*, 1995), microwave-enhanced anodic-stripping detection (Yu *et al.*, 2001), retrospective analysis (Eric *et al.*, 1007), sono electroanalysis (Alastair *et al.*, 2000), sono ASV detection method (Richard *et al.*, 1998) and others. However, these techniques demand specialized ionization energy systems and complicated voltametric modification techniques. The voltammetric utilizing devices are easy to use and inexpensive. Herein, various methods that have been developed include the nano hydroxamic acid carbon paste electrode (Tesfaye *et al.*, 1999), boron doped diamond electrode (Andrew *et al.*, 1999), and carbon paste polymer film electrodes (DomeÂnech *et al.*, 2000). Metal mercury (Hg) is also often used owing to its characteristic of forming amalgam. Methods include cathodic adsorptive-stripping voltammetry (Percio *et al.*, 2003), differential pulse adsorptive-stripping voltammetry using hanging mercury drop electrode (Bhim *et al.*, 2003), mercury film deposition on glassy carbon electrodes (Sandra *et al.*, 2004), and mercury film electrode (Jin *et al.*, 1997) often used in detecting heavy metals. Also, the carbon nanotube catalytic (Joseph *et al.*, 2004) structure is effective for bioassays (Marıa *at al.*, 2004). In this study, mercury and carbon nanotube paste were combined for lead detection. Optimized analytical result was attained to sensitive detection limits over other common methods, indicating that the method can be applied to plant and *in vivo* diagnostic analysis.

## MATERIALS AND METHODS

***Apparatus, reagents, HNPE preparation, and voltammetric producer.*** Electrochemical Workstation (660A CH Instruments Inc., Cordova, TN) was used for setting up the electrochemical system. A three electrode cell system was used in the cyclic and square-wave stripping voltammetry. Analytical *in vivo* assay was performed using the plant cell (Eichhornia crassipes 150 gram) and living fish (250 gram, a carp). A needle type, counter and reference electrode were inserted in the muscle and cells using a 0.5 mm micro diameter hand drill under anesthesia, 10~20 mm deep into the tissue. All the electrodes were cemented with a tooth binder and connected to a 0.05 mm enamel coated copper wire with an electrochemical system.

The modified HNPE working electrode (prepared using 40% metal Hg, 40% carbon nanotube, and 10% mineral oil mixed paste) and the PE (prepared using 90% carbon nanotube and 10% mineral oil mixed paste) were inserted in a 3 mm diameter glass tube. A reference electrode was used on an Ag/AgCl (saturated KCl) or chloride-coated 0.5-mm diameter silver wire-type electrode, whereas a 0.5-mm diameter platinum wire was used for the auxiliary electrode. Electrolyte solutions were prepared using 18 M-Ohm-cm$^{-1}$ double-distilled water. For the blank electrolyte solution, a 0.1 M ammonium phosphate buffer solution was used. The optimum pH strength was adjusted by adding 0.1 M HCl or a 0.1 M NaOH standard. Under these conditions, HNPE and PE were compared, and their concentration effects and optimum voltammetric parameters were sought.

## RESULTS AND DISCUSSION

***Cyclic comparison.*** Fig. 1. shows the comparison of the effects of HNPE and PE. First, the various electrolyte solutions were tested, with the ammonium phosphate buffer solution yielding good results. Under the aforementioned conditions, 1, 2, 3, 4, 5, 6, and 7 ppm Pb(II) standards were put in the ammonium phosphate buffer solution. The peak current of HNPE reached 0.7154, 0.6305, 1.514, 3.696, 5.98, 8.06, and 11.54 × $10^{-1}$ A, respectively. The peak current of PE went up to 0.1021, 0.3356, 1.094, 1.933, 2.721, 3.661, and 6.219 × $10^{-1}$ A, respectively, and HNPE's peak became

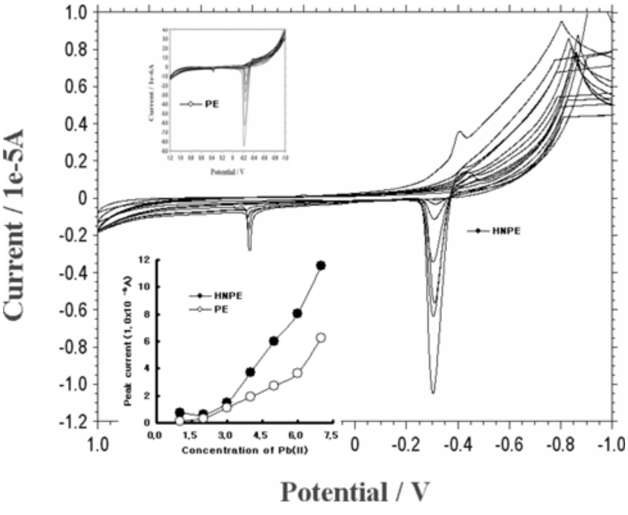

**Fig. 1.** Comparison of PE (insert voltammograms) and HNPE in three electrode cell systems within the 1~7 mg/l range (insert curves), using -1.0 V initial potential, 1.0 V switching potential, and 50 mV scan late for CV.

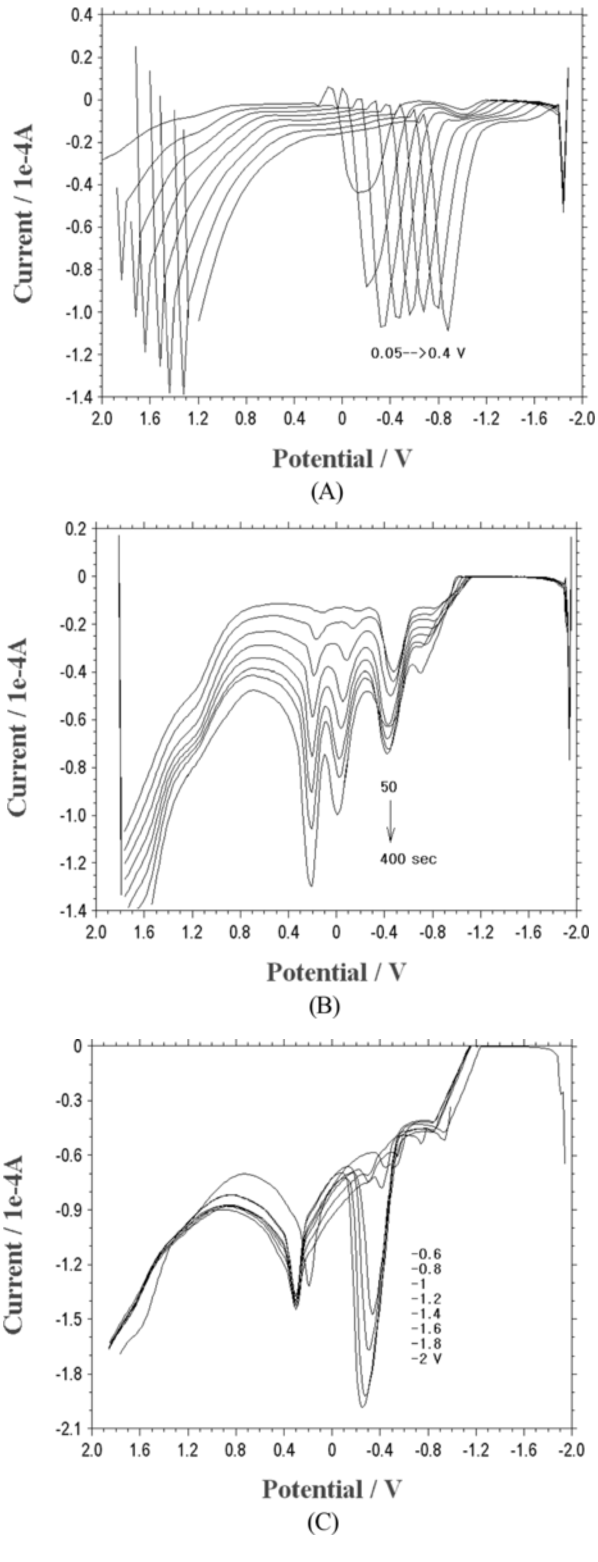

**Fig. 2.** Optimization of the SW stripping voltammetric parameters. (A) the amplitudes variation of 0.05, 0.1, 0.15, 0.2, 0.25, 0.3, 0.35, and 0.4 V. (B) the accumulation times 50, 100, 150, 200, 250, 300, 350, and 400 sec variation. (C) the accumulation potentials -0.6, -0.8, -1, -1.2, -1.4, -1.6, -1.8, and -2 V variation, using 4.0 pH electrolyte strength.

narrower and more sensitive. This means that HNPE is more sensitive to Pb(II) than PE. The oxidation peak current became more sensitive, instead of being reduced. As such, HNPE was chosen as the working electrode. Under anodic scan, the square wave oxidation stripping parameters were examined.

***Stripping voltammetric optimization.*** Fig. 2(A) shows the peak current in the 15 mg/l Pb(II) concentration as a function of varying square-wave amplitudes for the 0.05~0.4 V ranges. At 0.05 V amp, the stripping peak current of HNPE reached 4.761 × $10^{-1}$ A, but at 0.05, 0.1, 0.015, 0.02, 0.025, 0.03, 0.035, and 0.04 V amp, it rose to 9.062, 10.62, 9.904, 9.472, 9.43, 8.98, and 9.888 × $10^{-1}$ A. Here, the 0.04 V amp was sharp and sensitive; it was thus chosen as the optimized amplitude. Fig. 1(B) illustrates the accumulation times within the 50~400 s range. At 50 s, the stripping peak current of HNPE reached 3.603 × $10^{-1}$ A, and at 100, 150, 200, 250, 300, 350, and 400 s, it became 2.735, 3.375, 4.06, 4.234, 4.311, 4.295, and 2.855 × $10^{-1}$ A. 300 s was chosen as the optimized accumulation time. Under this condition, three peak potentials were obtained, but the -0.4 V peak was used. Fig. 1(C) shows various square-wave accumulation potentials for the -0.6~-2 V range. At -0.6 V, the stripping peak current of HNPE reached 0.44 × $10^{-1}$ A, and at -0.8, -1, -1.2, -1.4, -1.6, -1.8, and -2 V, it became 0.6009, 2.169, 8.662, 12.83, 10.92, 13.71, and 1.861 × $10^{-1}$ A. As such, -1.8 V was chosen as the optimized stripping accumulation potential. Under this condition, the other influence parameters of SW frequency, SW increment potential, and electrolyte hydrogen ionic strength were examined (not shown here). Optimum parameters were used for working curves and statistical application.

***Working range, statistics and application.*** After obtaining the optimum conditions, the working ranges were examined. Fig. 3(A) shows the resulting ppm range. When 0.8, 1.6, 2.4, 3.2, 4, 4.8, 5.6, and 6.4 mg/l were spiked, the peak current reached 0.7107, 2.506, 6.564, 10.85, 16.57, 20.12, and 25.47 × $10^{-5}$ A, respectively. The peak current appeared sharply at -0.2 V. The statistics of $y = 4.679x - 6.349$ and $R^2 = 0.956$ herein can have environmental applications. Fig. 3(B), on the other hand, shows the result of the micro ranges. When 10, 30, 50, 70, 90, 110, 130, 150, 170, and 190 ppb were spiked, they reached 1.784, 2.274, 3.892, 5.853, 8.57, 10.56, 11.66, 12.43, 12.65, and 11.91 × $10^{-1}$ A, respectively. The peak current also appeared sharply at -0.2 V. A linear curve was produced from the graph from a regression equation of $y = 0.802x + 0.4663$ (y: peak current in A; x: concentration in ppb) and a correlation of $R^2 = 0.9486$. This can be useful in biological assay and diagnostic *in vivo* detection, among others. Moreover, the ppb range is more sensitive that other photometric and separation methods. Thus, it can be applied to biological cell systems such as water, fish, plant tissue, and potato cells. Fig. 4(A) shows the SW result of the analytical application of HNPE to pond water. Each peak yielded an electrolyte blank, 1-ml pond water, and 1-, 3-, and 5 μg/; Pb(II) standards, respectively. In the 1-ml water sample, the SW reached 4.861 × $10^{-1}$ A, but at 1, 3, and 5 μg/l Pb spike, it went up to 4.861, 10.510, and 14.340 × $10^{-1}$ A. The result of

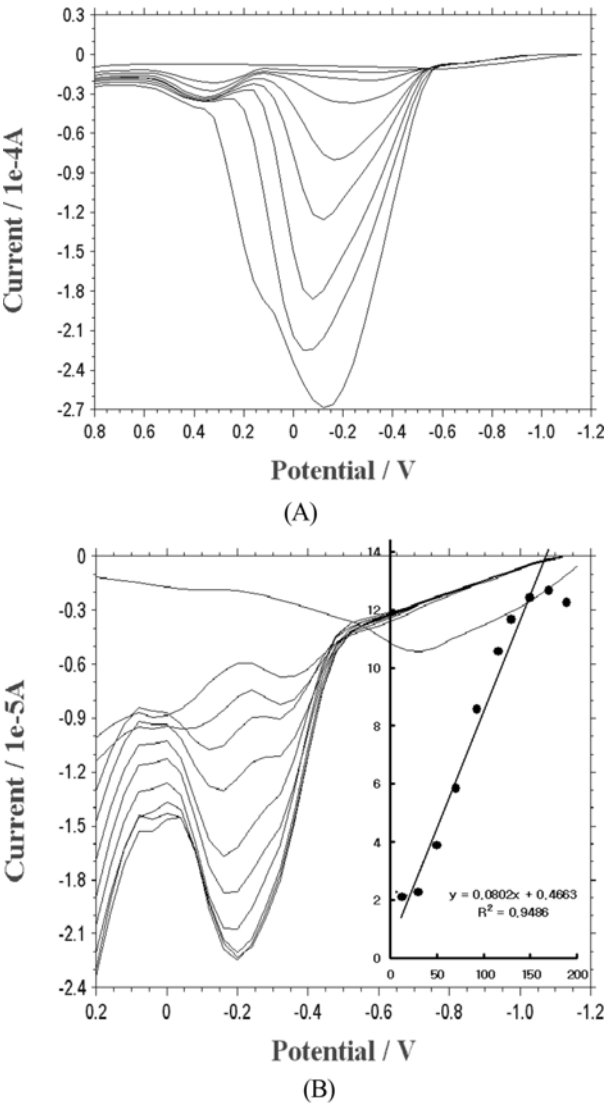

**Fig. 3.** (A) The result of anodic SW when 0.8- to 6.4 mg/l Pb(II) was added. (B) The result of anodic SW when 10- to 190-μg/l Pb(II) was added using the optimum parameters, and there working curve (insert linear equations).

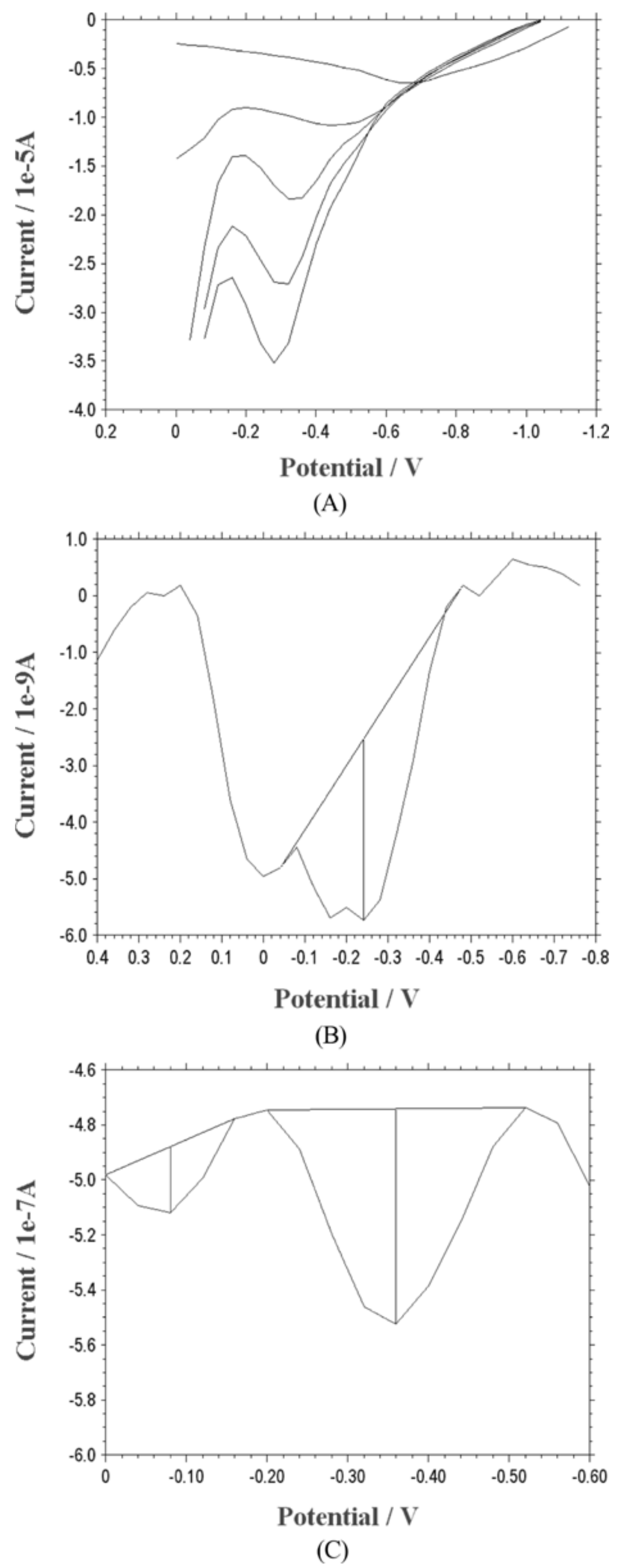

**Fig. 4.** The SW result of the application to (A) pond water by optimum conditions for 0.04 V amplitude, 400 Hz frequency, -8.0 V initial potential, 0.02 V increment potential and 300 sec accumulation times. (B) fish tissue and (C) plant tissue using the optimum conditions of *in vivo* inserting cell tissue.

4.52 μg/ml Pb(II) shows that it increased in terms of sensitivity scale. This means that HNPE can detect Pb within low working ranges. A more advanced application to a biological cell system was done. Fig. 4(B) shows the SW results of the analytical real-time application to fish tissue. A counter electrode (platinum), a reference electrode, and HNPE were inserted into a fish tissue, after which lead with HNPE was detected. The peak current appeared at -0.2 and -0.3 V Pb ion. Thus, Pb was found in the fish body. Fig. 4(C) shows the SW result of the analytical application of HNPE to plant tissue. Before the examination, the plant cell was contaminated using the 100 ppb Pb(II) spike in the plant water. Six hours later, three electrode systems were inserted into the plant cell tissue in a 5-mm dip. Under the cell tissue, the SW stripping voltammograms were scanned. The peak current also appeared at -0.2 and -0.3 V. This means that Pb(II) can be soaked in a plant. These developed techniques can be used for real-tissue assay and diagnostic analysis in *in vivo* cell systems.

## CONCLUSION

The optimal parameters of HNPE were found to be 0.04 V SW amplitude, 400 Hz frequency, -8 V initial potential, 0.02 V increment potential, 300 s accumulation time, and pH 4.0 strength. Under these conditions, HNPE was applied to the biological cell tissues of a fish, plant, and others. Pb(II) can also be soaked in plants via polluted water and detected direct. These results show that it is important to monitor lead, and that there is a need for a convenient lead detection method. The final method can easily detect even an infinitesimal trace range for in vivo and vitro diagnostics.

## ACKNOWLEDGEMENT

This work was supported by the Ministry of Land Transport and Maritime Affairs of Korea (grant PM55092).
